# Assessment of depression in total laryngectomy patients

**DOI:** 10.1016/j.bjorl.2025.101677

**Published:** 2025-08-05

**Authors:** Kelen Fernanda Catarochi, Vaneli Colombo Rossi, Carlos Takahiro Chone

**Affiliations:** Universidade Estadual de Campinas, Departamento de Otorrinolaringologia Cirurgia de Cabeça e Pescoço, São Paulo, SP, Brazil

**Keywords:** Laryngectomy, Depression, Total laryngectomy, Quality of life, Cancer

## Abstract

**Objective:**

Of this study was to evaluate the presence and severity of depressive symptoms in total laryngectomy patients and identify variations in results of each item on the depression scale.

**Methods:**

This study is a quantitative, analytical and cross-sectional study, with 30 total laryngectomized participants, using the Instrument (BDI-II) Beck Depression Inventory-II, from April to July 2024, all total laryngectomized participants who attended the Head and Neck surgery Otorhinolaryngology Outpatient Clinic and were invited to participate in the research, the assessment was carried out by the same reserarcher and the estimated response time was 22 min.

**Results:**

Show tha depression was identified in 20% of patients with mild symptoms, 13% moderate and 20% severe. The sleep pattern was altered in 63% of respondents and difficulty concentrating was reported by 57%. Altered appetit was identified in 53%. The feeling of sadness was identified in 60% of patients, also crying, pessimism and loss of interest in 40%. Regarding irritability, agitation and feelings of punishment, 47% of participants showed changes. The self-criticism and self-esteem were preserved in 70% and 63% respectively. Suicidal thoughts or desires were identified in 17% and any percentage for this resuts needs attention. The group was 80% male, the average age of participants is 65±7-years. Married patientes are 67%, divorced 20%, singles 3% and widower are 10%. The time after surgery ranged fron 1 to 10 years; 50% had undergone chemotherapy and 73% denied post-surgical complications.

**Conclusion:**

This this study points to is that 53% of patients showed signs of depression on some scale.

**Level of evidence:**

Level 3.

## Highlights

• Symptoms of depression are found in a large percentage of total laryngectomy.
• Sleep patterns show significant changes in 63% of the cases interviewed.
• Marital status, sex, age and therapies do not correlate with depression.
• The longer the time after surgery, the lower the depression scale index.

## Introduction

A total laryngectomy is the surgical removal of the larynx, a procedure first performed by Christian Albert Theodor Billroth in Vienna in 1873. Since then, advanced laryngeal and hypopharyngeal cancers have been the main indications for laryngectomy.^1^

Globally, almost 200,000 cases of laryngeal cancer are diagnosed each year.^2^ It is more common in men. Risk factors include alcohol and tobacco consumption, viral infections, reflux, environmental influences, and genetic factors such as the Human Papillomavirus (HPV). Symptoms such as hoarseness, dyspnea, and dysphagia should be considered warning signs. Persistent hoarseness lasting more than four weeks and leukoplakia are early indicators; 20% of patients develop laryngeal cancer within five years.

A total laryngectomy patient faces a life-altering mutilation process, with profound effects caused by anatomical and physiological changes that affect the loss of voice, lung function, swallowing, and sense of smell. Although surgery may be indicated for oncological cure and safety of dysfunctional larynges, patients may experience a significant loss of desire to live. Clear information about the procedure's impacts, including its effects on partners or family members, is important. Women tend to have greater difficulty with marital intimacy and often reject the new voice.^3^

The electrolarynx and esophageal voice yield poorer speech outcomes compared to patients using the tracheoesophageal prosthesis.^4^

In a group of 35 patients who underwent laryngeal surgery, approximately one-third exhibited clinically significant symptoms of depression and/or anxiety, negatively impacting their quality of life.^5^

The quality of life of laryngectomy patients depends on the success of rehabilitation regarding voice, feeding, and social interaction.^6^ In France, a study with 133 patients aged 60 to –81 showed that 8% returned to some form of work, one-third reported being frightened by the tracheostomy, and many felt poorly informed about how the voice prosthesis works. The lack of information leads to isolation and insecurity, and 73% considered receiving educational support to be useful.

Through voice prostheses, it is possible to achieve better voice quality, though they require daily maintenance and can cause complications. Filters and adhesives help regulate the heat and humidity of tracheal respiration, minimizing tracheobronchial problems. However, these resources are not always accessible due to delays in fitting the prosthesis or lack of financial resources. An individual’s self-identity is closely linked to communication, and contact with other laryngectomy patients can be beneficial. Returning to work is difficult, and medical follow-up must be regular.^7^

The aerodigestive tract undergoes changes due to the lack of continuity between the nasopharynx and the lower airway, with laryngectomy patients depending on the stoma for breathing. As a result, decreased stimulation of the olfactory bulb, atrophy of the nasal epithelium, and destruction of ciliated cells collectively contribute to olfactory dysfunction and nasosinus disease. Thirty percent of these patients report a negative impact on their olfactory quality of life, while 63% report negative outcomes regarding nasosinus-specific quality of life, with significantly worse results for nasal congestion, rhinorrhea, post-nasal drip, and olfaction.^8^

In Australia, a study of 113 participants revealed a significant reduction in physical health-related quality of life and social relationships, with higher levels of depression and anxiety. Speech and swallowing impairments were mild compared to psychological well-being.^9^

In a comparison of quality of life versus longevity, both were considered equally important.^10^ However, in cases of advanced cancer, quality of life was prioritized over longevity; this group wanted to maintain their activities and not become a burden on their families. They chose treatment only if they believed the prognosis could improve.

After laryngectomy, the overall health of these patients can be considered only “satisfactory”, as social restrictions, physical and mental stress, reduced olfaction, an altered voice, insecurity in personal behavior, dry mouth, cough, fatigue, shortness of breath, insomnia, financial difficulties, and fears about the future emerge. Psychosocial well-being often falls well below expected levels.^2^

In a study involving 172 laryngectomy patients, this population scored lower on all quality-of-life scales, with 7% to –9% being categorized with some degree of depression and 12% to 8% with anxiety.^11^

In Japan, 150 laryngectomy patients were studied for anxiety, depression, and quality of life in the pre-operative and post-operative phases over 60 months. The study showed that, in all quality-of-life measures, results were significantly worse compared to the general population at the beginning and three months after surgery. However, general health, vitality, mental health, and bodily pain improved to normal levels within one year after surgery and remained stable at five years. Thirty-five percent were categorized as potential cases of depression, and 35% as potential cases of anxiety.^12^

Dysphagia occurs in approximately 90% of total laryngectomy cases, leading to long and exhausting meals, food limitations, weight loss, malnutrition, decreased psychological well-being, distress, and impaired social interactions. This condition leads patients to eat in isolation and lose the pleasure of eating, with significant emotional consequences. In Paraíba, Brazil, a study of 14 total laryngectomy patients suggested that mental disorders and swallowing difficulties are not related in the long term. However, the number of psychological symptoms increases in patients with swallowing complaints.^13^

According to the National Cancer Institute of Brazil,^14^ the estimated number of new cases of laryngeal cancer in Brazil is 7,790 per year, with 6,570 in men and 1,220 in women, ranking 18th among the most frequent cancer types.

It is common for laryngectomy patients to present depressive symptoms. However, diagnosing depression is challenging, as it can be confused with side effects of treatment or natural responses to life-threatening situations and pain. In order to formally assess depressive symptoms regularly and throughout the treatment trajectory, standardized and validated measures for oncology populations are used, including the Beck Depression Inventory II (BDI-II).^15^

The BDI-II version was developed and updated by Beck, Steer, and Brown. The version used was adapted to Brazilian Portuguese.^16^ This instrument is internationally recognized in the scientific community for assessing the presence and intensity of symptoms.

In the book On Death and Dying,^17^ Kübler-Ross offers a humanized view of facing death, exploring the emotions and experiences of terminally ill patients and their caregivers. This perspective also applies to total laryngectomy patients, as they undergo an intense process of life redefinition. The author highlights the importance of listening, embracing, and respecting what each patient experiences during this process, emphasizing that open communication and emotional support are essential to bring dignity to those facing illness. Kübler-Ross introduces the five stages of grief: denial, anger, bargaining, depression, and acceptance.

In psychoanalysis, mourning is understood as a psychological process necessary to deal with the loss of an object of affection, whether it be a person, a condition in life, or even an idea. Freud, in his essay Mourning and Melancholia (1917),^18^ describes mourning as a psychic work in which the individual must confront the reality of the loss. This implies a painful detachment from the emotional bond with the lost object and finding new meaning in the condition to which the individual is subjected. This process involves reliving memories and emotions associated with the loss, allowing the mourner to redirect psychic energy, previously focused on the object or idea.

This study aims to analyze the presence and severity of depressive symptoms in patients who have undergone total laryngectomy and to identify variations in the results of each item on the BDI-II depression scale.

## Methods

This is a quantitative, analytical, cross-sectional study, based on groups of patients who underwent total laryngectomy at a reference center in Campinas, Brazil.

The research was approved by the Human Research Ethics Committee of Unicamp, CAAE: [7620.1920.0.0000.5404]; Opinion No. [6.764.841].

A convenience sample was used. All consecutive patients from the population of total laryngectomy patients who attended medical, dental, speech therapy, psychology, physical therapy consultations, nursing team procedures, or any other intervention at the Otorhinolaryngology and Head and Neck Surgery Outpatient Clinic Unicamp were invited to participate in the study.

Data collection occurred from April to July 2024, with participants aged 18 and older, with no exclusion based on the time since surgery.

Participants received explanations about the Informed Consent Form (ICF), the BDI-II application procedure, and the estimated time for completion.

The Beck Depression Inventory-II (BDI-II) assessment was administered by a single researcher, following the same application standards and criteria, individually, in a quiet, closed room with no noise or interruptions. The BDI-II is a self-administered instrument consisting of 21 items designed to measure the intensity of depression in individuals aged 13 and older. These items include sadness, pessimism, sense of failure, dissatisfaction, guilt, punishment, self-confidence, self-blame, suicidal thoughts, crying, irritability, social withdrawal, indecision, self-worth, loss of energy, sleep disturbance, appetite change, concentration difficulties, somatic concerns, and loss of libido.^16^

Instructions to participants followed a standardized procedure.

Frequency tables were calculated for categorical variables and measures of central tendency and dispersion for continuous variables, including age, gender, sex, years post-surgery, chemotherapy, radiotherapy, post-surgical complications, and depression outcomes, including the degree of depression or absence of depression.

The statistical tests used were Fisher’s exact test, Mann-Whitney or Kruskal-Wallis tests, and Spearman’s correlation coefficient. The significance level was set at 5%.

## Results

Data from 30 participants were collected, excluding one individual aged 13 who did not meet the established age criteria for the study.

Among the 30 patients studied:

• The average age was 65.5-years (±7.0-years), with a minimum age of 49 and a maximum of 80-years.
• The sample consisted of 80% men and 20% women.
• Regarding marital status: 66.7% were married, 20% divorced, 3.3% single, and 10% widowed.
• Half of the patients (50%) underwent chemotherapy after surgery, while 76.6% received radiotherapy.
• Post-surgical complications were denied by 73% of the patients.
• The average time after surgery ranged from 1 to 10-years.
• Analysis of depressive symptoms using the BDI-II revealed:
• Sadness: 60% of participants reported some degree of sadness (50% mild, 3.3% moderate, 6.7% severe).
• Difficulty concentrating: 57% reported some level of impairment (43.3% mild, 13.3% moderate).
• Sleep disturbances: 63% of patients experienced negatively altered sleep patterns.
• Fatigue: 63% reported fatigue.
• Appetite changes: 53% reported either increased or decreased appetite.
• Lack of energy: 53% reported low energy levels.
• Crying: 47% reported crying more than usual.
• Feelings of guilt and irritability: Both observed in 47% of participants.
• Agitation: 47% of patients showed signs of emotional disorganization.
• Loss of sexual interest: Also reported by 47%.

Regarding other BDI-II items:

• 50% of patients showed significant results related to devaluation (sense of usefulness or uselessness), indecision, and loss of pleasure in activities.
• For loss of interest, past failure, and pessimism, 40% showed depressive indicators, while 60% maintained a healthy perception.
• Self-esteem: preserved in 70% of patients.
• Self-criticism: preserved in 63.3% of patients.
• Suicidal thoughts:
• 83% denied any suicidal ideation.
• 13.3% reported suicidal thoughts without intent to act.
• 3.3% expressed a desire for suicide but no intent to act.

General depressive outcomes:

• 53% of patients exhibited depressive symptoms:
• 20% mild;
• 13% moderate;
• 20% severe.
• 46.7% did not show depressive symptoms.

Statistical analyses showed:

• The longer the time since surgery, the lower the depression scores;
• No significant differences were found regarding sex or age.
• Comparative analysis using Fisher’s exact test, Mann-Whitney test, and Chi-Square showed no significant differences between the groups with and without depressive symptoms regarding demographic variables.

## Discussion

The results of this study reveal a significant prevalence of depressive symptoms in patients undergoing total laryngectomy, with 53% of participants presenting some degree of depression according to the Beck Depression Inventory II (BDI-II). These findings corroborate previous studies that highlight depression as a common comorbidity among these patients.^9^

The high prevalence of significant sadness (60%) and the presence of other depressive symptoms, such as difficulty concentrating (57%) and altered sleep patterns (63%), may be indicators of psychological impact. Studies suggest that the loss of voice, a direct consequence of laryngectomy, can trigger deep feelings of loss and social isolation, which are known risk factors for depression. The quality of life for these patients depends significantly on the quality of rehabilitation and adaptation to a new living condition.^7^

Additionally, the high frequency of fatigue (63%) and lack of energy (53%) observed in participants may be associated not only with the psychological impact of the surgery but also with physical factors, such as dysphagia,^13^ and adjuvant treatments with chemotherapy and radiotherapy, performed by 50% and 76.6% of patients, respectively. Literature indicates that oncological treatments, especially when combined, can exacerbate fatigue symptoms and contribute to the physical and mental exhaustion of patients.^19^

Most cancer patients prioritize quality of life over longevity; patients state that they wish to maintain their activities and avoid becoming a burden on their families.^10^

The analysis also revealed that feelings of devaluation, indecision, and loss of pleasure were present in 50% of the patients, highlighting the need for targeted psychological interventions. Psychological support, with strategies for giving patients space to express and process their new life condition, is effective in reducing depressive symptoms and improving quality of life.^14^

A relevant aspect of the findings is the preservation of self-esteem and self-criticism in a significant portion of patients (70% and 63.3%, respectively), suggesting that despite emotional challenges, many patients maintain a positive self-perception. This may indicate the presence of resilience factors, such as social support,^20^ which were not directly evaluated in this study but deserve attention in future research.

The low incidence of suicidal thoughts (13.3%) is a positive finding. However, the presence of such thoughts in any percentage of patients is concerning and requires constant monitoring and preventive interventions. Regular assessment of mental health and the provision of psychological support are crucial to minimize emotional impacts.^15^

It is important to recognize that depressive symptoms in laryngectomy patients can overlap with side effects of treatment, requiring careful clinical evaluation for proper management.

Some limitations of this study should be considered:

• Although the sample size (n = 30) is significant for this population, it limits the generalization of the results.
• The absence of a control group prevents direct comparison with patients who did not undergo total laryngectomy.
• The study did not explore in-depth factors such as social support, socioeconomic status, and pre-existing comorbidities, which may influence the results.

Future studies should consider these aspects for a more comprehensive understanding of depression in laryngectomy patients.

## Conclusion

The assessment of depression in patients who underwent total laryngectomy revealed a significant presence of depressive symptoms, with 53% of participants showing some degree of depression according to the BDI-II scale. Furthermore, the severity of depressive symptoms tends to decrease with the increase in time after surgery, indicating a possible psychological adaptation.

No significant differences in depression levels were observed between different genders or age groups in the analyses.

These findings highlight the importance of implementing continuous psychological support strategies for laryngectomized patients, with a focus on early interventions and adjustments over time to enhance the overall quality of life and emotional well-being of this population.

## ORCID ID

Kelen Fernanda Catarochi: 0002-8336-7416

Vaneli Colombo Rossi: 0000-0002-7930-8838

Carlos Takahiro Chone: 0000-0002-4217-4629

## Funding

There was no source of funding for this research.

## Declaration of competing interest

There are no conflicts of interest with the participants or any other collaborators, whether direct or indirect, in the development of this Research Project.
