# Supplementary material for: Assessment of depression in total laryngectomy patients
Source: Braz J Otorhinolaryngol. 2025 Aug 5;91(6):101677. doi: 10.1016/j.bjorl.2025.101677 (PMC12624774; doi:10.1016/j.bjorl.2025.101677)
Supplement: Supplementary file 1 [file mmc1.docx]

**BJORL-D-25-00054_Supplementary Material**

**Table Supplementary 1** Statistical result with variables.

| **Sex** | | | |
| --- | --- | --- | --- |
| Feminine | 20.00% | 6 |  |
| Maculine | 80.00% | 24 |  |
| **Marital status** | | | |
| Married | 66.70% | 20 |  |
| Divorced | 20.00% | 6 |  |
| Single | 3.30% | 1 |  |
| Widower | 10.00% | 3 |  |
| **Chemotherapy** | | | |
| Yes | 50.00% | 15 |  |
| No | 50.00% | 15 |  |
| **Radiotherapy** | | | |
| Yes | 76.70% | 23 |  |
| No | 23.30% | 7 |  |
| **Post surgical complication** | | | |
| Sim | 26.70% | 8 |  |
| Não | 73.30% | 22 |  |
| **Average age** | | |  |
| 7 anos +- | 58 | 65 | 72 |
| Min / Max | 49 | 65 | 80 |
| **Years after surgery** | | | |
| Years | 1.3 | 5.4 | 10.1 |

**Table Supplementary 2** Variables of the statistical results of each BDI-II.

| **Sadness** |  |  |
| --- | --- | --- |
| Minimum | 40.00% | 12 |
| Light | 50.00% | 15 |
| Moderate | 3.30% | 1 |
| Serious | 6.70% | 2 |
| Total depression | 60% | 18 |
| **Pessimism** |  |  |
| Minimum | 60.00% | 18 |
| Light | 26.70% | 8 |
| Moderate | 6.70% | 2 |
| Serious | 6.70% | 2 |
| Total depression | 40% | 12 |
| **Past failure** |  |  |
| Minimum | 60.00% | 18 |
| Light | 13.30% | 4 |
| Moderate | 20.00% | 6 |
| Serious | 6.70% | 2 |
| Total depression | 40% | 12 |
| **Lost of pleasure** |  |  |
| Minimum | 50.00% | 15 |
| Light | 20.00% | 6 |
| Moderate | 20.00% | 6 |
| Serious | 10.00% | 3 |
| Total depression | 50% | 15 |
| **Feeling of guilt** |  |  |
| Minimum | 53.30% | 16 |
| Light | 33.30% | 10 |
| Moderate | 10.00% | 3 |
| Serious | 3.30% | 1 |
| Total depression | 47% | 14 |
| **Feeling of punishment** |  |  |
| Minimum | 53.30% | 16 |
| Light | 33.30% | 10 |
| Moderate | 10.00% | 3 |
| Serious | 3.30% | 1 |
| Total depression | 47% | 14 |
| **Self esteem** |  |  |
| Minimum | 70.00% | 21 |
| Light | 16.70% | 5 |
| Moderate | 13.30% | 4 |
| Serious | 0.00% | 0 |
| Total depression | 30% | 9 |
| **Self review** |  |  |
| Minimum | 63.30% | 19 |
| Light | 23.30% | 7 |
| Moderate | 13.30% | 4 |
| Serious | 0.00% | 0 |
| Total depression | 37% | 11 |
| **Suicidal thoughts or desires** |  | |
| Minimum | 83.30% | 25 |
| Light | 13.30% | 4 |
| Moderate | 3.30% | 1 |
| Serious | 0.00% | 0 |
| Total depression | 17% | 5 |
| **CRY** |  |  |
| Minimum | 60.00% | 18 |
| Light | 13.30% | 4 |
| Moderate | 20.00% | 6 |
| Serious | 6.70% | 2 |
| Total depression | 40% | 12 |
| **Agitation** |  |  |
| Minimum | 53.30% | 16 |
| Light | 30.00% | 9 |
| Moderate | 13.30% | 4 |
| Serious | 3.30% | 1 |
| Total depression | 47% | 14 |
| **Lost of interest** |  |  |
| Minimum | 60.00% | 18 |
| Light | 10.00% | 3 |
| Moderate | 23.30% | 7 |
| Serious | 6.70% | 2 |
| Total depression | 40% | 12 |
| **Indecision** |  |  |
| Minimum | 50.00% | 15 |
| Light | 16.70% | 5 |
| Moderate | 20.00% | 6 |
| Serious | 13.30% | 4 |
| Total depression | 50% | 15 |
| **Desvaluation** |  |  |
| Minimum | 50.00% | 15 |
| Light | 20.00% | 6 |
| Moderate | 23.30% | 7 |
| Serious | 6.70% | 2 |
| Total depression | 50% | 15 |
| **Lack of energy** |  |  |
| Minimum | 46.70% | 14 |
| Light | 20.00% | 6 |
| Moderate | 23.30% | 7 |
| Serious | 10.00% | 3 |
| Total depression | 53% | 16 |
| **Change in sleep pattern** |  | |
| Minimum | 36.70% | 11 |
| Light | 33.30% | 10 |
| Moderate | 23.30% | 7 |
| Serious | 6.70% | 2 |
| Total depression | 63% | 19 |
| **Irritability** |  |  |
| Minimum | 53.30% | 16 |
| Light | 30.00% | 9 |
| Moderate | 10.00% | 3 |
| Serious | 6.70% | 2 |
| Total depression | 47% | 14 |
| **Change in appetite parttern** |  | |
| Minimum | 46.70% | 14 |
| Light | 23.30% | 7 |
| Moderate | 16.70% | 5 |
| Grave | 13.30% | 4 |
| Total depression | 53% | 16 |
| **Difficulty concentrating** |  | |
| Minimum | 43.30% | 13 |
| Light | 43.30% | 13 |
| Moderate | 13.30% | 4 |
| Serious | 0.00% | 0 |
| Total depression | 57% | 17 |
| **Tiredness or fatigue** |  |  |
| Minimum | 36.70% | 11 |
| Light | 36.70% | 11 |
| Moderate | 13.30% | 4 |
| Serious | 13.30% | 4 |
| Total depression | 63% | 19 |
| **Loss of interest in sex** |  | |
| Minimum | 53.30% | 16 |
| Light | 23.30% | 7 |
| Moderate | 16.70% | 5 |
| Serious | 6.70% | 2 |
| Total depression | 47% | 14 |
| **Total result** |  |  |
| Minimum | 46.70% | 14 |
| Light | 20.00% | 6 |
| Moderate | 13.30% | 4 |
| Serious | 20.00% | 6 |
| Total depression | 53% | 16 |

**Table Supplementary 3** Relationship between the categorized result and variables.

|  | **Serious** | **Light** | **No Depression** | **Moderate** | **Total** |  |
| --- | --- | --- | --- | --- | --- | --- |
| Variable | (n = 6) | (n = 6) | (n = 14) | (n = 4) | (n = 30) | p-valor |
| **Years after surgery** | | | | | | |
| Minimum |  |  |  |  |  |  |
| Maximum | 3.8 ± 2.4 | 6.5 ± 5.3 | 6.9 ± 4.9 | 3.0 ± 1.2 | 5.7 ± 4.4 | 0.0877¹ |
| Median | 4.0 (1.0-8.0) | 5.0 (2.0-17.0) | 6.0 (3.0-22.0) | 3.0 (2.0-4.0) | 4.5 (1.0-22.0) |  |
| **Years** | | | | | | |
| Minimum – máximum | 68.2 ± 6.1 | 63.0 ± 10.3 | 66.4 ± 6.5 | 62.3 ± 3.3 | 65.5 ± 7.0 | 0.4086¹ |
| Median | 67.0 (62.-76) | 61.5(49.-75.) | 66.0 (54.0-80) | 62.5 (58.0-66.0) | 65.0(49-80) |  |
| **Sex** | | | | | | |
| F | 2 (33.3%) | 1 (16.7%) | 2 (14.3%) | 1 (25.0%) | 6 (20.0%) | 0.8510³ |
| M | 4 (66.7%) | 5 (83.3%) | 12 (85.7%) | 3 (75.0%) | 24 (80.0%) |  |
| Total | 6 | 6 | 14 | 4 | 30 |  |
| **Marital status** | | | | | | |
| Married | 4 (66.7%) | 3 (50.0%) | 11 (78.6%) | 2 (50.0%) | 20 (66.7%) | 0.5367³ |
| Others | 2 (33.3%) | 3 (50.0%) | 3 (21.4%) | 2 (50.0%) | 10 (33.3%) |  |
| Total | 6 | 6 | 14 | 4 | 30 |  |

^1^ Test Kruskal-Wallis test.

³ Fisher's exact test.

**Table Supplementary 4** Relationship between numerical result BDI-II and sex marital status.

| Sex | N | Mean | Std Dev | Minimum | Median | Max. (Mann-Whitney) | |
| --- | --- | --- | --- | --- | --- | --- | --- |
| F | 6 | 20.00 | 17.19 | 0.00 | 21.00 | 44.00 | 0.5158 |
| M | 24 | 14.00 | 11.12 | 0.00 | 13.00 | 40,00 |  |
| Marital status | N | Mean | Std Dev | Minimum | Median | Max. (Mann-Whitney) | |
| Married | 20 | 14.15 | 11.89 | 0.00 | 11.00 | 40.00 | 0.6275 |
| Others | 10 | 17.30 | 13.93 | 0.00 | 17.50 | 44.00 |  |

**Table Supplementary 5** Correlation between numerical BDI-II results and yers after surgery/age.

| Spearman Correlation Coefficient, n = 30 (2) p-value | | | |
| --- | --- | --- | --- |
|  | | | |
|  | **Total** |  |  |
| Years after surgery | -0.41838 | -1 | Longer the time |
| Years after surgery | 0.0214 | -2 | Lower the score |
|  | | | |
| Years | 0.09739 | -1 |  |
| Years | 0.6087 | -2 |  |

**Table Supplementary 6** Variables with results fromthe BDI-II instrument

| Result total = no depression / with depression (min.light.moderate) | | | | |
| --- | --- | --- | --- | --- |
| **Variable** | **(n = 16) With depression** | **(n = 14) No depression** | **Total**  **(n = 30)** | **p-value** |
|  |  |  |  |  |
| **Years after surgery** | | | | |
| Median | 4.6 ± 3.7 | 6.9 ± 4.9 | 5.7 ± 4.4 | 0.0565¹ |
| Minimum/Max | 1.0 - 17.0 | 3.0 - 22.0 | 1.0 - 22.0 |  |
|  |  |  |  |  |
| **Age** | | | | |
| Median | 64.8 ± 7.6 | 66.4 ± 6.5 | 65.5 ± 7.0 | 0.4279¹ |
| Minimum/Max | 49.0 - 76.0 | 54.0 - 80.0 | 49.0 - 80.0 |  |
|  |  |  |  |  |
| **Sex** | | | | |
| F | 4 (25.0%) | 2 (14.3%) | 6 (20.0%) | 0.6567³ |
| M | 12 (75.0%) | 12 (85.7%) | 24 (80.0%) |  |
| Total | 16 | 14 | 30 |  |
|  |  |  |  |  |
| **Marital status** | | | | |
| Casado | 9 (56.3%) | 11 (78.6%) | 20 (66.7%) | 0.2602³ |
| Outros | 7 (43.8%) | 3 (21.4%) | 10 (33.3%) |  |
| Total | 16 | 14 | 30 |  |
|  |  |  |  |  |
| **Chemotherapy** | | | | |
| No | 9 (56.3%) | 6 (42.9%) | 15 (50.0%) | 0.4642² |
| Yes | 7 (43.8%) | 8 (57.1%) | 15 (50.0%) |  |
| Total | 16 | 14 | 30 |  |
|  |  |  |  |  |
| **Radiotherapy** | | | | |
| No | 6 (37.5%) | 1 (7.1%) | 7 (23.3%) | 0.0860³ |
| Yes | 10 (62.5%) | 13 (92.9%) | 23 (76.7%) |  |
| Total | 16 | 14 | 30 |  |
|  |  |  |  |  |
| **Post surgical complication** | | | | |
| No | 10 (62.5%) | 12 (85.7%) | 22 (73.3%) | 0.2255³ |
| Yes | 6 (37.5%) | 2 (14.3%) | 8 (26.7%) |  |
| Total | 16 | 14 | 30 |  |
